# Impact of Morphometry, Myelinization and Synaptic Current Strength on Spike Conduction in Human and Cat Spiral Ganglion Neurons

**DOI:** 10.1371/journal.pone.0079256

**Published:** 2013-11-08

**Authors:** Frank Rattay, Thomas Potrusil, Cornelia Wenger, Andrew K. Wise, Rudolf Glueckert, Anneliese Schrott-Fischer

**Affiliations:** 1 Institute for Analysis and Scientific Computing, Vienna University of Technology, Vienna, Austria; 2 Department of Otorhinolaryngology, Innsbruck Medical University, Innsbruck, Austria; 3 Faculty of Informatics, Vienna University of Technology, Vienna, Austria; 4 Institute of Biophysics and Biomedical Engeneering, Faculty of Science, University of Lisbon, Lisbon, Portugal; 5 The Bionics Institute, East Melbourne, Australia; 6 University Clinics Innsbruck, Tiroler Landeskrankenanstalten, Innsbruck, Austria; Georgia State University, United States of America

## Abstract

**Background:**

Our knowledge about the neural code in the auditory nerve is based to a large extent on experiments on cats. Several anatomical differences between auditory neurons in human and cat are expected to lead to functional differences in speed and safety of spike conduction.

**Methodology/Principal Findings:**

Confocal microscopy was used to systematically evaluate peripheral and central process diameters, commonness of myelination and morphology of spiral ganglion neurons (SGNs) along the cochlea of three human and three cats. Based on these morphometric data, model analysis reveales that spike conduction in SGNs is characterized by four phases: a postsynaptic delay, constant velocity in the peripheral process, a presomatic delay and constant velocity in the central process. The majority of SGNs are type I, connecting the inner hair cells with the brainstem. In contrast to those of humans, type I neurons of the cat are entirely myelinated. Biophysical model evaluation showed delayed and weak spikes in the human soma region as a consequence of a lack of myelin. The simulated spike conduction times are in accordance with normal interwave latencies from auditory brainstem response recordings from man and cat. Simulated 400 pA postsynaptic currents from inner hair cell ribbon synapses were 15 times above threshold. They enforced quick and synchronous spiking. Both of these properties were not present in type II cells as they receive fewer and much weaker (^∼^26 pA) synaptic stimuli.

**Conclusions/Significance:**

Wasting synaptic energy boosts spike initiation, which guarantees the rapid transmission of temporal fine structure of auditory signals. However, a lack of myelin in the soma regions of human type I neurons causes a large delay in spike conduction in comparison with cat neurons. The absent myelin, in combination with a longer peripheral process, causes quantitative differences of temporal parameters in the electrically stimulated human cochlea compared to the cat cochlea.

## Introduction

The temporal characteristics of the spiking patterns of the auditory nerve are crucial in natural hearing [1]–[4] and in its neuroprosthetic counterpart when action potentials (APs) are initiated via cochlear implants [5]–[8]. Most of our knowledge on spike coding in the auditory nerve is based on single cell recordings in cats. As such recordings are not possible in man, findings from cats are often generalized to human due anatomical similarities. However, shorter total lengths of SGNs in cat, thinner processes, smaller cell bodies and fundamental differences in myelination are obvious reasons not to rely on a cat model when signaling in human auditory nerve is discussed as these differences between the species may lead to important differences in auditory nerve function.

**Figure 1:**
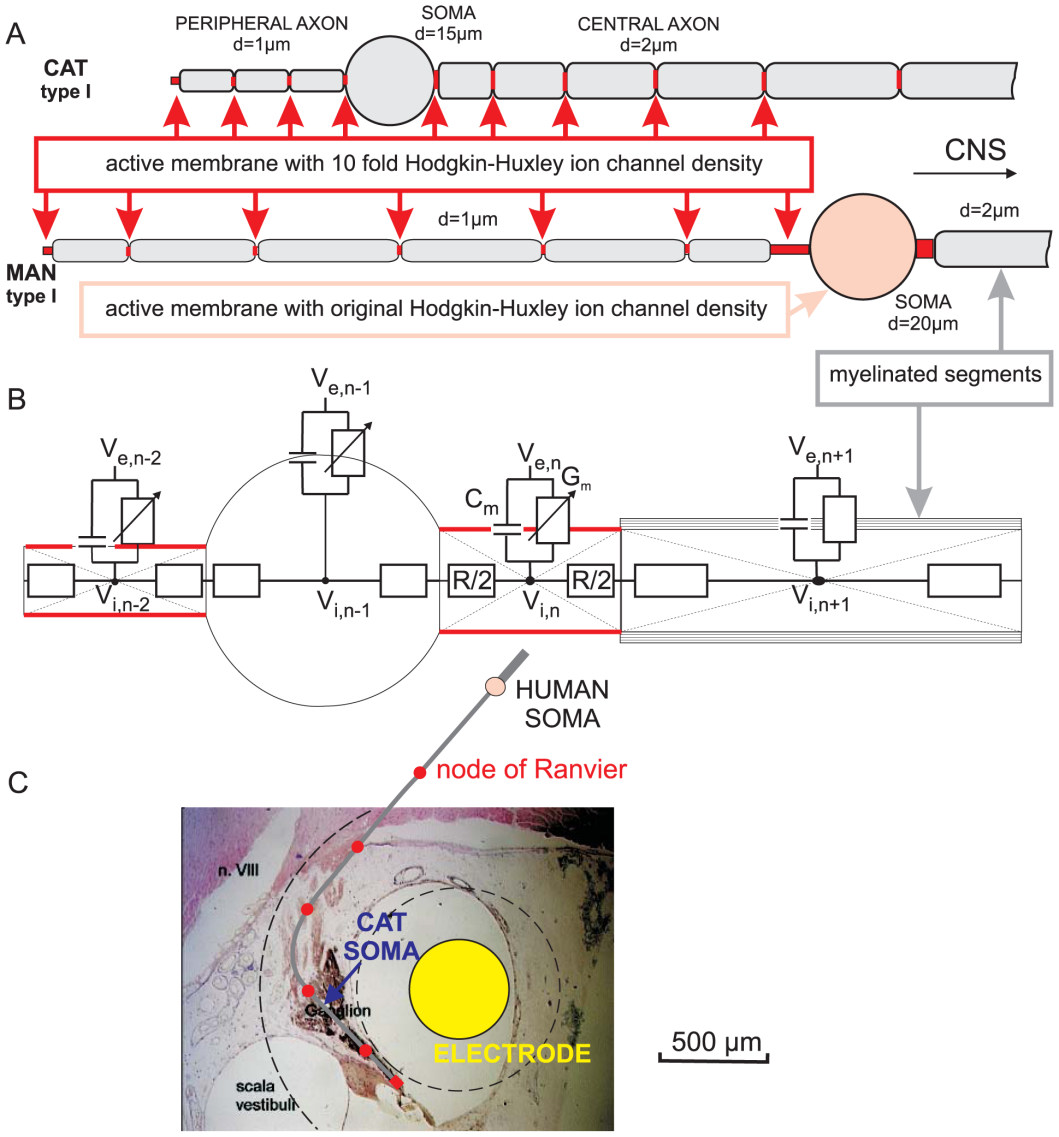
Compartment models for SGNs. (A) Type I cells, rectified: Myelinated segments are shown in gray. Excitable (active) membranes with high ion channel densities (red segments) in the peripheral terminal and in the nodes of Ranvier are needed for spike amplification. In contrast to feline cells, in man the pre- and postsomatic compartments are longer, the soma is larger and not myelinated and the peripheral as well as the central axons are longer. (B) According to Ohm's law the sum of all currents to the center of a compartment is zero. The currents are defined by extracellular potential V_e_, intracellular potential V_i_, membrane capacitance C_m_, membrane conductance G_m_ and intracellular resistance R. Natural excitation by synaptic current from a hair cell ribbon synapse is simulated as current injection into the first compartment (peripheral terminal). In this case extracellular potentials V_e_ are assumed to be zero. For nonmyelinated type II cells the same modeling approach was used with uniform ion channel densities as in the original Hodgkin-Huxley model and with constant compartment lengths in the axons. (C) The same neural pathway of a human type I cell model as used in [7] is placed over a cross section of a feline cochlea demonstrating a possible position of a scala tympani electrode relative to a target cell. The length relations are the same as in the rectified versions in A. Extracellular potentials are calculated for a homogeneous infinite medium which causes spherical isopotentials, indicated by dashed lines. Note that the cat soma is much closer to the electrode than the human one.

In the cochlea, sensory hair cells convert sound into neural signals. These afferent signals are conducted along the auditory nerve by two types of SGNs. The vast majority of SGNs are bipolar type I cells with large cell bodies connecting inner hair cells (IHCs) with the cochlear nuclei, whereas smaller type II neurons transmit APs from the outer hair cells (OHCs) [9], [10]. Both types of cochlear neurons constitute the primary afferent input to the cochlear nucleus [11], [12] in the brainstem which represents the first central relay station in the ascending auditory pathway [13]. Type II neurons are usually completely unmyelinated and have similar physiological properties in man and cat. However, standard type I cochlear neurons in humans are crucially different compared to that of other mammalians as their cell bodies as well as the pre- and post somatic segments lack myelin [14]–[16](Figure 1A). These human differences, together with varying diameters of the peripheral- and central processes [17], [18], which determine AP velocities, suggest a diversity in auditory signal conduction times along SGNs between man and cat.

The high conduction velocities of myelinated axons are based on the electrical properties of the internode, which is the part between two nodes of Ranvier. Assuming a conducted AP in an idealized internode (membrane capacity C = 0, membrane conductance Gm = 0) would cause the same temporal characteristics in the transmembrane voltage profile at both ends, i.e. the conducted AP is reduced in amplitude by intracellular resistance but the maxima at the beginning and end of the internode appear at the same time. This phenomenon, known as saltatory conduction [19], requires only a very small capacitance. This is reached by many circumjacent myelin layers as C is inversely proportional to the number of myelin sheets covering the cells membranes [20]. A spike propagation problem may arise in SGNs when the small inneraxonal current of the thin peripheral process has to load the large capacitance of an unmyelinated soma [7], [21], which represents the main barrier for an AP along the neural tract.

A previously presented compartment model for cochlear neurons [7] was evaluated in this study in order to analyze the impact of myelination and morphometry on temporal features of signal conduction in cat and man. Important parameters for the velocity of spike conduction are the AP duration and its rise time. Different types of voltage-gated ion channels have been identified in SGN membranes based on rat and mouse data [21], [22], some with impact on the falling part of the spike or on accommodation and refractoriness [23]. However most ion channel models for non-myelinated neural membrane segments in warm blooded animals, including one of SGN type II of mouse [21], show AP durations longer than 1 ms [24], [25]. An example for shorter APs is the octopus cell in the cochlear nucleus [26] whose spikes are still of double duration of a SGN.

In contrast to such membrane models, the original Hodgkin-Huxley model with a temperature fit to 27°C resulted in APs with a duration of 330 µs and a short rise time that was consistent with intracochlearly recorded SGN spikes of cats [7], [27], [28]. In this paper we present the first modeling study that analyzes the temporal features of spike conduction along afferent SGNs of man and cat. Immunohistochemistry and confocal microscopy imaging was performed to distinguish type I and II neurons and quantify process diameters of bipolar SGNs as well as myelination in cat and human cochleae. Major differences concerning the extent of myelination, morphometric characteristics and the length of the neural path between these two species were incorporated in our SGN model. In addition, computer simulation enables the analysis of functional effects of synaptic current strengths that differ essentially between type I and II neurons [29], [30].

## Materials and Methods

### Ethics Statement

Human temporal bones were obtained during routine autopsy at the Institute of Pathology, Innsbruck Medical University, Austria. According to the Tiroler Krankenanstaltengesetz § 37 - Tir KAG, from Aug 14, 2013, consent is not required for the use of autopsy samples, and so informed consent requirement was waived. No individually identifiable patient data is presented in this report, and all data presented is therefore anonymous. Reevaluation from archival celloidine and plastic embedded sections emanate from previous research projects and were published by Spoendlin and others [31], [32]. All procedures for animal tissue were approved by the Royal Victorian Eye and Ear Hospital Animal Research & Ethics Committee.

### Specimens

The study is based on three human temporal bones from individuals aged 56 to 74 without any diagnosed ear disease or hearing loss (audiograms were not available). Human cochleae were fixed with 4% paraformaldehyde for 24 h at 4°C, subsequently decalcified in 20% ethylenediaminetetraacetic acid at pH 7.4 for 6 weeks and prepared for cryoembedding according to [33]. Details are described [34]. Inner ears were serially sectioned with a cryomicrotome perpendicular and radial to the modiolus at 10 µm and 35 µm respectively.

Three young adult cats (8 – 10 months old, 3.5 – 4.5 kg sourced from the BRC located at Royal Victorian Eye and Ear Hospital) were deeply anesthetized (sodium pentobarbitone; Nembutal; i.p., 60 mg/kg) and intracardially perfused with physiological saline (37°C) containing heparin (0.1% v/v) and sodium nitrate (0.025% v/v), followed by 10% neutral buffered formalin (NBF) at 4°C. The bullae were then removed and carefully opened to access the cochlea. Round and oval window were perforated with a microneedle and the cochleae were locally perfused with 10% NBF. Cochleae were post fixed with NBF for 1 hr at room temperature, then washed in PBS and stored in PBS (with 0.003% Sodium Azide).

Measurements of unmyelinated peripheral- and central process diameters were carried out on three male, normal hearing human subjects (49 – 63 year old) without any diagnosed ear disease (audiograms not available) from archival material. Evaluation of cat process diameters are based on three inner ears from young female adult individuals, one year old, taken from archival material. Human as well as cat cochleae were fixed perilymphatically with ice-cold Karnovsky's solution and prepared using a standard celloidin embedding technique [35]. Specimens were serially sectioned perpendicularly to the modiolus at 25 µm thickness and stained with hematoxylin and eosin.

In order to quantify the length of a standard bipolar SGN used in our computational model, we analyzed CT/MR data acquired during daily clinical practice of three male human individuals (aged 14 to 46 years) and three adult domestic cats (2–3 years old).

### Immunohistochemistry

Staining was performed after washing the sections in phosphate-buffered saline (PBS, pH = 7.4, 6×5 min) followed by blocking of unspecific reactions with PBS containing 5% bovine serum albumin (BSA, Serva, Germany), 15% non-immune normal donkey serum (NDS, Chemicon-Merck, Austria) and 0.3% Triton X-100 (Sigma, Germany) for 2 h at room temperature (RT). Primary antibodies (Table 1) were diluted with a solution containing PBS, 1.67% BSA, 5% NDS and 0.1% Triton X-100. Cryosections were subsequently incubated for triple staining in a humid, darkened chamber at 4°C overnight followed by 1 h 15 min incubation at 37°C. After rinsing with PBS (6×5min) sections were incubated with secondary antibodies conjugated to Alexa Fluor™ 488 (donkey anti-rabbit, 1∶1000, Invitrogen), Alexa Fluor™ 546 (donkey anti-mouse, 1∶1000, Invitrogen) and Alexa Fluor™ 633 (goat anti-rat, 1∶1000, Invitrogen) diluted with PBS for 2 h at RT. After rinsing the stained sections with PBS (6×5 min) cell nuclei were counterstained using 4′,6-diamidino-2-phenylindole, dihydrochloride (DAPI, 1∶46000, Molecular Probes) for 35 min at RT. Sections were mounted after rinsing with PBS (6×5 min) using Vectashield™ mounting medium for fluorescence microscopy.

**Table 1:** Primary antibodies for immunohistochemistry.

antibody	dilution	characteristics	host	source
anti-β-III-tubuline	1∶500	monoclonal	mouse	Chemicon, MAB5544
Anti-myelin basic protein	1∶100	monoclonal	rat	Sigma-Aldrich, M9434
anti-peripherin	1∶2500	polyclonal	rabbit	Millipore, AB1530
4′,6-diamidino-2-phenylindole, dihydrochloride	1∶46000			Molecular Probes, D1306

### Electron microscopy

Human specimens were prepared according the block surface technique described by Spoendlin and Brunn [36] and Spoendlin and Schrott [37]. Subsequently, a Zeiss® Libra 120 (Zeiss®, Oberkochen, Germany) transmission electron microscope (University of Innsbruck, Institute of Zoology and CMBI) operating at 80 kV with 500 ms exposure time was used for examining ultra-thin sections at magnification of 200×. Digital images were captured using a TRS 2048 HSC High Quality camera.

### Confocal imaging and processing

Immunostained sections were imaged using a Zeiss LSM 510 Meta confocal laser-scanning microscope equipped with a 20×/0.8 NA dry lens and a 63×/1.4 NA oil immersion lens. To simultaneously detect cell nuclei, type II neurons, cell bodies and SGN myelin we used a 405 nm diode laser, a 488 nm line of an argon-krypton laser, a 543 nm and a 633 nm HeNe laser. Image capturing was performed using ZEN® software (Zeiss, Jena, Germany).

To measure process diameters, three-dimensional image stacks were acquired from the celloidin embedded sections using a 63×/1.4 NA oil immersion lens and a 488 nm argon ion laser combined with a 505 nm long-pass filter. The emitted blue-green light was used to leverage the autofluorescence of the hematoxylin and eosin stained SGNs. Pixels sizes of the 3D-stacks were selected according to the Nyquist theorem resulting in the following resolution: x = 43 nm, y = 43 nm, z = 130 nm. In order to reduce the distortion created by the microscope and increase the quality of the quantitative analysis of the 3D-stacks, a theoretical point spread function was calculated for each image channel. These point spread functions were subsequently used to deconvolute the acquired image stacks using a non-blind maximum-likelihood image restoration algorithm [38] over 40 iterations.

Diameters were evaluated from the unmyelinated pre- and postsomatic segments of SGNs located in the Rosenthal's canal of the apical, middle and basal regions of the samples.

### Measurement of nerve length

Each CT/MR data pair was registered using normalized mutual information method [39]. Fused data was used to manually segment the brainstem and the cochlear nerve from the right and left cochlea (starting point for length measurement was defined in the middle of the cochlea at the height of the middle turn) via the internal auditory meatus to the cochlear nucleus of each individual. Visualizing the brainstem by surface rendering enabled us to determine the position of the VIII^th^ nerve entering the brainstem (endpoint for length measurement). This enabled the calculation of the total length of cochlear nerve fibers as cochlear nuclei are located next to this entry point.

In order to calculate the lengths of the manually segmented nerves, the xyz-coordinates of the highlighted pixels at each DICOM slice were extracted using Fiji Win64bit image processing software [40] using a macro to highlight a single pixel and save its respective coordinates from every image of the analyzed stack. Each line segment was considered as a vector. The sum of their lengths resulted in the total nerve length of the analyzed human and cat specimens.

Medical imaging data and acquired confocal image stacks were processed with a high-performance workstation (Z800, Hewlett-Packard, Palo Alto, CA, USA) using ImageJ v1.44 and Amira 5.4 (Mercury Computer Systems Inc., San Diego, CA, USA). 3D-data from celloidin embedded sections were processed as described previously [34].

### Statistical analysis

Statistical significance was determined by one-way analysis of variance followed by Bonferroni correction. Quantile-quantile plots were calculated to assess normality of data. Descriptive statistics, significance of data as well as the actual power for the sample data (significance level  = 0.01) were computed with Matlab® 2011a (MathWorks, Natick, MS, USA).

### Computer simulation

We used the same compartment model, suited for human and cat, as described in detail in [7] but changed geometrical parameters as well as the numbers of shielding myelin layers according to new morphometric and immunohistochemical evaluations. Accordingly, each neuron was split up into compartments, either with an active membrane with original Hodgkin Huxley ion channel kinetics at 27°C, or as passive internodes (Figure 1A). Transmembrane resistances r_m_ of internodes and myelinated somas were assumed to be constant and proportional to the number N of myelin layers (r_m_ = N*1 kOhm/cm^2^) [7]. The spiking of a target neuron was simulated by an electrical network, where the currents to the center of each compartment consist of a resistive and a capacitive current across the membrane as well as intracellular currents to the neighboring compartments (Figure 1B). The temporal profile of the transmembrane voltage of each compartment was computed by solving a system of four differential equations for compartments with an active membrane and by a single differential equation for the internode. Current injection into the first compartment of the model neuron, that is the peripheral terminal, simulated the synaptic activation by a hair cell.

The standard model for a human type I SGN consisted of a myelinated somatic region with few membrane layers that separate the heavily myelinated peripheral and central processes. This structure was modeled as the following sequence of compartments: an unmyelinated distal terminal, followed by an internode and 5 node-internode combinations, a presomatic region (segmented into three compartments for computational accuracy), soma, postsomatic region, internode-node combinations of the central process and finally an unmyelinated central terminal. The standard value used for the inner diameter of the peripheral process was 1 µm. According to our morphometric findings, the central process diameter was always of double size. Soma was assumed as a sphere with 20 µm in diameter, all other parameters as in [7]. The somatic region was assumed to be covered by three membrane layers. The standard model for a cat type I cell differed from the human case in relation to fewer and shorter internodes, lack of pre- and postsomatic unmyelinated segments, smaller soma diameter (15 µm) with 13 shielding myelin sheets covering the soma (Figure 1A). The standard model for unmyelinated type II SGNs had the same process diameters as the myelinated ones for man and cat, but for computational accuracy more and shorter axonal compartments. For details about geometric and electrical parameters see [7] methodic and basic concepts are found in [20], [41], [42].

## Results

The first part of this work depicts the results of the immunohistochemical analysis of various geometrical parameters and the occurrence of myelination among human and cat SGNs. The collected data were subsequently incorporated in our cochlear neuron model to test their impact on spike excitation and signal conduction.

### Myelination and soma sizes of SGNs in cat and man

#### Cat

**Figure 2:**
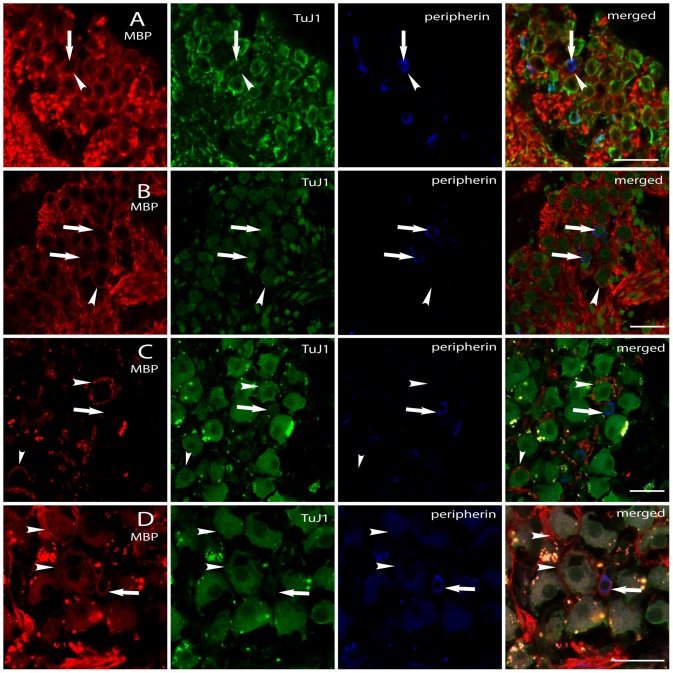
Immunofluorescence of MBP, TuJ1 and peripherin in cat (A, B) and human (C, D) spiral ganglion. (A, middle turn region) shows a myelinated type II neuron in a cat inner ear. The arrow highlights the peripherin positive cell body of a type II cochlear neuron; the arrow head depicts its isolating myelin. (B, basal region) illustrates two unmyelinated type II neurons (white arrows) which were (partly) surrounded by myelin of neighboring type I neurons. Additionally, the arrow head points to a type I SGN which was fully surrounded by myelin. Note the absence of these surrounding myelin layers at the depicted type II neurons. A myelinated type I neuron (white arrow head) and an unmyelinated type II neuron (white arrow) of the human spiral ganglion are shown in (C, basal turn). D (middle turn) presents a human type II cell body surrounded by myelin (white arrow) and type I neurons surrounded by myelin (white arrow heads). Scale bars 30 µm.

In cat cochleae we found that in 95.54% of neurons (Table 2) examined both the soma and the processes of type I SGNs (n = 3229) were surrounded by soma myelinating satellite glial cells (myelinating SGCs) within the spiral canal and myelinating Schwann cells within the osseous spiral lamina. Both types of glial cells were positive for myelin basic protein (MBP). The myelinating SGCs ensheath the entire cell body (Figure 2A/B, MBP) forming the distinct honeycomb structure in Rosenthal's canal sections where the cell bodies of the SGNs reside.

**Table 2:** Summary of the detected myelinated and type II spiral ganglion cells.

	cat cochlea (n = 3)				human cochlea (n = 3)			
cochlea region	n =	myel. cell	type II	d [µm]	n =	myel. cell	type II	d [µm]
basal	1234	1186	50	10.25±0.91	1109	36	16	9.36±0.70
percentage %		96.11	4.05			3.25	1.44	
middle	995	942	55	9.72±1.16	981	42	29	9.52±0.86
percentage %		94.67	5.53			4.28	2.96	
apical	1000	957	42	9.82±0.82	893	31	15	9.18±0.80
percentage %		95.70	4.20			3.47	1.68	
total	3229	3085	147	9.93±1.01	2983	109	60	9.39±0.82
percentage %		95.54	4.55			3.65	2.01	

Presented are the total numbers of counted myelinated type I SGN and type II SGN somata from cat and human cochleae, their percentage as well as the evaluated soma diameters. In contrast to man, the vast majority of cell bodies analyzed from cat cochleae were found to be myelinated.

Type II cochlear neurons were identified using an anti-peripherin antibody. This neuron-specific intermediate filament protein is well known to be expressed in mammalian (including human) type II SGNs [43]–[47] enabling the identification of these small cochlear neurons using confocal microscopy. The middle cochlear regions showed highest density of peripherin positive type II neurons (5.53% of n = 995 analyzed neurons) followed by the apical (4.2% of n = 1000) and basal (4.05% of n = 1234 cells) regions. A total number of 147 cells (Table 2) were identified in cat cochleae as type II neurons, being positive for the peripherin protein (Figure 2A & B). This value represents 4.55% of all analyzed cat neurons. Moreover, type I and type II neurons of cats stained positive for TuJ1 protein (Figure 2 A, B, TuJ1).

Four type II SGNs (2.72%, n = 147) were found to have fully myelinated cell bodies (Figure 2A). However, the vast majority of peripherin protein positive type II cells (97.28%) feature unmyelinated cell bodies (Figure 2B) as well as unmyelinated peripheral and central processes. One 3D-image stack of such a standard type II neuron was acquired during data analysis. The measured peripheral- and central process diameter of this neuron was d1 = 0.73 µm and d2 = 1.3 µm respectively, and was subsequently used in our computational model for further analysis.

**Figure 3:**
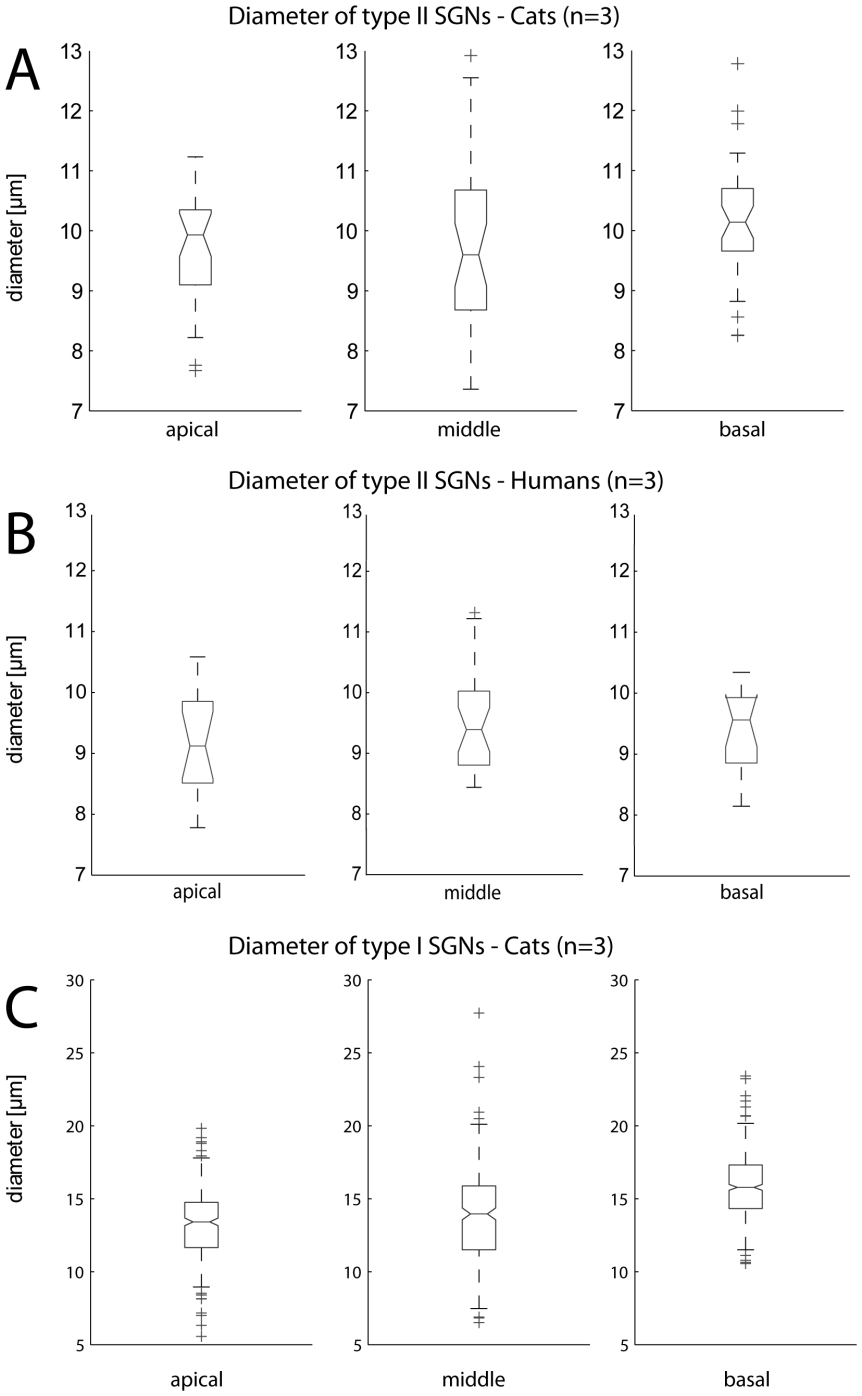
Box-Whisker-Plot depicting variations of type II SGN soma diameters for (A) cat and (B) man according to their specific region within the inner ear. (C) presents the evaluated diameters of type I SGN somata from the analyzed cat specimens located in the apical, middle and basal region of the cochleae.

The diameters of peripherin positive type II neurons were measured from deconvoluted 3D image stacks. Scrolling through the z-stack enabled the detection of the maximum diameter of the evaluated cell bodies, which was measured and used for statistical analysis. Type II neurons had a uniform soma diameter across all cochlear regions (Figure 3A) with a maximum in the basal region (median (M)  = 10.29 µm), the smallest values with the largest variance (M = 9.68 µm) in the middle turn and M = 9.92 µm in the apical region.

All three analyzed cat specimens showed the highest proportion of SGNs that are type II neurons within the middle turn region reaching 5.44% (n = 515) in specimen 1, 6.61% (n = 121) in specimen 2 and 5.29% (n = 359) in specimen 3. The smallest percentages of type II cells were identified in the apical regions of specimen 1 and 3 (3.85%, n = 182; 4.2%, n = 262) whereas the minimum appeared in the basal region of specimen 2 (3.73%, n = 322). Notable is the size distribution of type II SGNs, where the largest cell bodies were found in the high frequency region in all analyzed specimens.

The largest type I SGNs were identified in the basal regions (Figure 3C) of the analyzed cat specimens with a mean soma diameter of 15.81±2.03 µm (n = 537). Type I SGNs located in the middle turn reached a mean diameter of 13.74±3.25 µm (n = 284). The apical regions of the investigated cat cochleae contained the smallest type I neurons with a measured mean diameter of 13.41±2.29 µm (n = 427). Cell bodies of type I neurons located in the apical- and middle turn were found to be significantly smaller (p<0.01, power = 1) compared to those from the basal region. We used an average diameter of 15 µm of these types of SGNs in our computer simulations since the mean diameter of all analyzed somas was determined to be 14.52±2.69 µm (n = 1248).

#### Human

Analyzing data from three individual human cochleae, we found that 3.65% of n = 2983 type I neurons (Table 2) were surrounded by myelinating SGCs strongly positive for MBP (Figure 2 C, D). The acquired confocal stacks revealed that the isolating myelin layers fully wrap around the cell somas without any gaps. Furthermore, the pre- and postsomatic segments of SGNs were similarly surrounded by myelin comparable to these neuronal units of other mammalians. The white arrowheads in Figure 2C highlight the MBP positive myelin layers of human type I SGNs. The merged image illustrates the myelinated cell bodies of human type I SGNs (TuJ1 positive) in contrast to the majority of unmyelinated SGNs.

The highest density of myelinated somas of n = 981 type I SGNs (4.28%) were identified in the middle turn, the minimum in the basal turns (3.25% of n = 1109 type I cells) and 3.47% of all analyzed cochlear type I neurons (n = 893) in the low frequency regions. The standard case of a human type II neuron (Figure 2C) has a fully unmyelinated cell body. Confocal 3D-stacks also showed the complete absence of myelin at the peripheral and central neurite (data not shown). In contrast to type II cells of cat, human type II SGNs were not positive for TuJ1 – protein. Solely type I - SGNs are strongly positive for this neuron-specific class III beta-tubulin.

A total number of 60 peripherin positive SGNs (Table 2) were identified representing 2.01% of all analyzed human neurons (n = 2983). The highest incidence of type II cells was found in the middle turn (2.96%), followed by apical turns (1.68%) and the high frequency basal regions (1.44%). Interestingly, two type II neurons representing 3.34% (n = 60) were found to be surrounded by myelinating SGCs (strongly MBP positive) wrapping myelin around the cell body as well as their processes. Again, the white arrow (Figure 2D) depicts a type II cell body positive for the peripherin protein. The merged image illustrates this cell and its surrounding myelin (red color). Additionally, white arrow heads point to myelinated type I cell bodies (Figure 2D). However, the standard type II neuron in the human inner ear completely lacks myelin. 3D image acquisition was performed on one type II cell with an unmyelinated cell body and processes. The evaluated process diameters used in our computational model are d1 = 0.65 µm and d2 = 1.4 µm. The median diameters of human type II somata from all specimens (Figure 3B) were relatively constant with a maximum (M = 9.55 µm) found in the high frequency basal region, M = 9.39 µm in the middle turn and the smallest value (M = 9.13 µm) in the apical region. The calculated mean values in table 2 identifies the largest diameters of type II cells in the middle turns, a region of prime importance for speech recognition [48]. However, no significant differences were found comparing type II cell soma diameters between cats and humans (p>0.05, power = 0.82).

**Figure 4:**
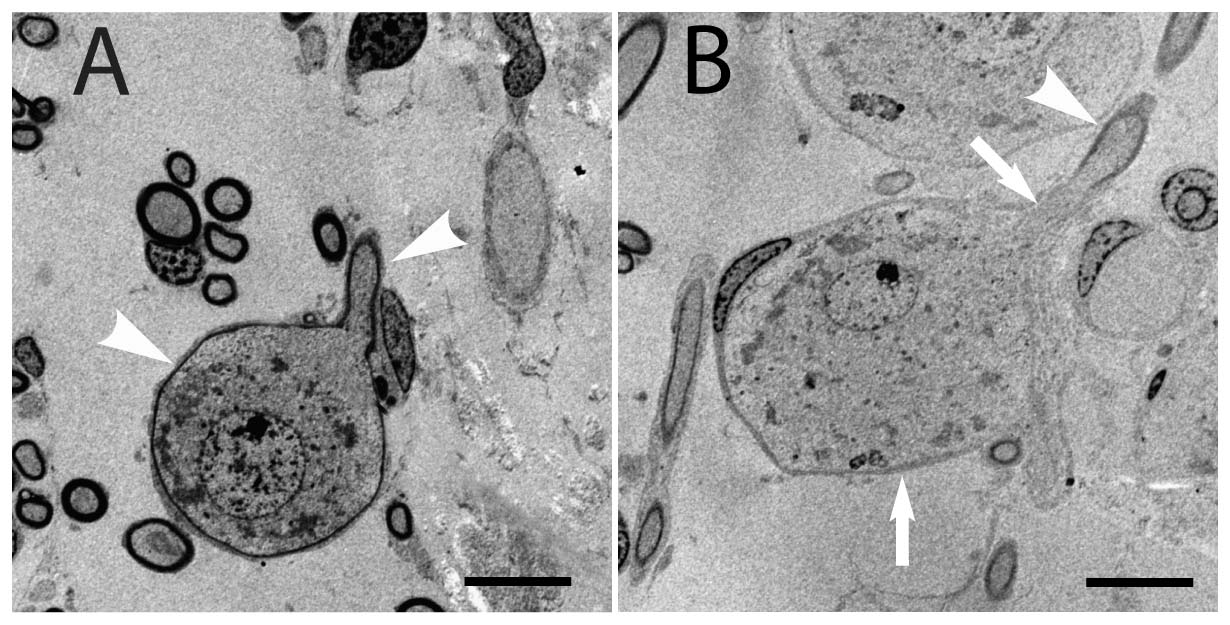
Transmission electron microscopy images of human SGNs. (A) Cell body of a putative type I SGN completely enwrapped with myelin. Additionally, the process of the SGN shows continuous myelination (white arrow heads). The standard human SGN is shown in B. White arrows highlight an unmyelinated cell body encircled by a satellite glial cell and the myelin lacking process of a SGN. The myelination of the central process starts after about 7 µm pointed by the white arrow head. Scale bar 10 µm.

The myelination of human SGNs was additionally assessed on electron microscopy level (Figure 4A). White arrow heads depict the myelinated cell body of a type I SGN cell body and the myelinated process of a neurite. Note the continuous myelination of cell body and neurite. Figure 4B shows the standard case of a large unmyelinated type I cell body (white arrows) that is covered by non-myelinating SGCs. The part of the central neurite that connects directly to the cell body totally lacks myelin. Myelination of the neurite starts here after approximately 7 µm (highlighted by the white arrow head) away from the soma.

Individual differences in incidence, myelination, soma- and neurite diameter in type II neurons are summarized as followed (data not shown). The middle turn of specimen 1 and specimen 2 contained the highest densities of type II neurons (1.96% of n = 255 and 1.46% of n = 274 analyzed cells respectively) as well as myelinated SGNs (4.71% and 5.11% respectively). In specimen 3, the maximum density of type II cells (5.41% of n = 148 evaluated neurons) and myelinated cells (5.41%) were both located within the apical regions. A surprising observation was the complete absence of type II neurons in the basal region of specimen 2 (n = 351 cochlear neurons) and its rare appearance in the basal (0.77% of n = 260 cells) and apical region (0.69% of n = 433 analyzed bipolar neurons) of specimen 1.

### SGN lengths in human and cat

**Figure 5:**
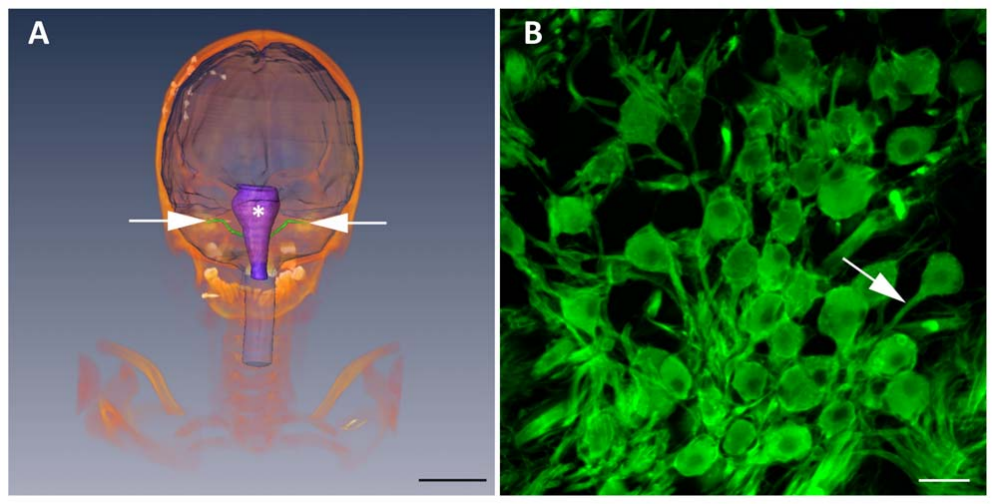
Visualization of SGN length measurement (A) and z-projection of a confocal image stack (B) of basal human cochlear neurons. (A) presents the volume rendered bone of an analyzed individual. The brain is illustrated in a transparent manner (blue) together with the manually segmented brainstem (white star). The starting points of the SGNs from the left and right cochleae are highlighted by the white arrows. The manually segmented Nervuli cochlearum are visualized using surface rendering (green colored). The white arrow in (B) highlights a central process connecting the cell body with the cochlear nucleus. The diameter of this neurite was measured to be 2.54 µm. Scale bar in (A) indicates 5 cm; in (B) it indicates 20 µm.

In order to compute afferent spike conduction times with the cochlear neuron model, we needed the lengths of cochlear nerve processes. By analyzing clinical computer tomography data (one example is presented in Figure 5A) of three human individuals (n = 6; right and left ear), we determined an average SGN length of 32.35±1.45 mm. Evaluation of manually segmented right and left auditory nerves of three domestic cats (n = 6) resulted in an average length of 15.81±0.39 mm, which is half the length compared to human cochlear neurons. For better comparison of spike conduction times, the lengths of type I and II cochlear neurons were assumed to be the same.

### Type I process diameters of SGNs in human and cat

**Figure 6:**
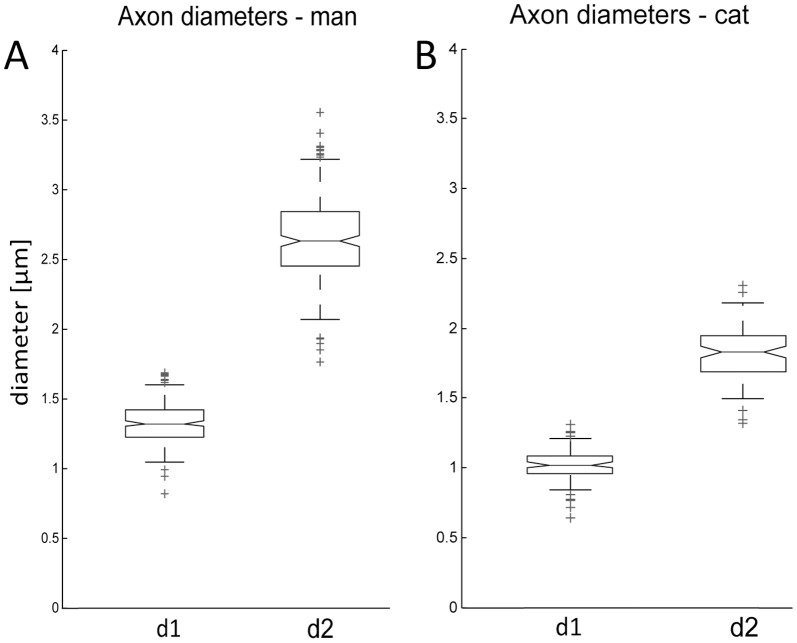
Box-Whisker-Plot illustrating measured peripheral- ( = d1) and central ( = d2) process diameters of type I SGN for man (A, n = 3) and cat (B, n = 3) within the inner ear.

Peripheral and central process diameters d1 and d2 were systematically evaluated within the Rosenthal's canal of three human specimens, which is illustrated in Figure 5B. The mean diameter (Table 3) of the peripheral processes (n = 212) was determined to be 1.32±0.15 µm. The central process, which transmits the AP from the cell body to the cochlear nuclei, was measured to be 2.65±0.3 µm (n = 236), which is double the size of the peripheral processes. Figure 6A illustrates the measured variations for d1 and d2 for human type I neurons. Note that deviations of about ±32% from the mean values for d1 and d2 were found. A closer evaluation of the different regions of the cochlea demonstrated no significant difference in process diameters comparing the basal-, middle- and apical region (Table 3, p>0.05, power = 1). Furthermore, each specimen was statistically analyzed according to these frequency specific regions as well as among themselves (data not shown). However, no significant differences regarding the diameters were found (p>0.05, power = 1).

**Table 3:** Peripheral and central process diameters of type I neurons based on 3 human specimens and 3 cat specimens.

human (n = 3)			cat (n = 3)	
region	d1 [µm]	d2 [µm]	d1 [µm]	d2 [µm]
basal	1.35±0.15 (n = 110)	2.67±0.29 (n = 125)	1.03±0.13 (n = 55)	1.8±0.2 (n = 59)
middle	1.28±0.13 (n = 66)	2.63±0.29 (n = 71)	0.99±0.11 (n = 22)	1.8±0.17 (n = 21)
apical	1.32±0.17 (n = 36)	2.60±0.34 (n = 40)	1.06±0.1 (n = 16)	1.88±0.15 (n = 13)
**total**	**1.32±0.15 (n = 212)**	**2.65±0.30 (n = 236)**	**1.02±0.12 (n = 93)**	**1.81±0.19 (n = 93)**

Data according to their location along the cochlea spiral.

Table 3 illustrates the findings of process diameters d1 and d2 from three cat cochleae. Data is presented for SGN processes without a myelin sheet. For this purpose, the diameters of myelinated fibers were measured in a first step and recalculated using an inner/outer fiber diameter ratio d/D = 0.7. The thinner peripheral process had a mean value of 1.02±0.12 µm (n = 93) whereas the mean diameter of the central process was 1.81±0.19 µm. Deviations from the mean of about ±30% were observed for d1 and d2 in cats (Figure 6B). Analyzing the interspecies differences between d1 of human and cat as well as d2 between these two species reveal to be highly significant for both measures (p<0.001, power  = 1).

### Computer simulation

In the first section, the temporal model features are evaluated with intracochlear recorded neural responses. Then, signal propagation in SGNs is shown to consist of four characteristic phases. Formulas for velocity-diameter relationships are found for the myelinated and unmyelinated axons of type I and type II cells. Systematic variations of model parameters show their impact on the total SGN signal conduction times for cat and man. In the last section consequences of strong postsynaptic IHC currents and weak postsynaptic OHC currents are analyzed.

### AP duration

**Figure 7:**
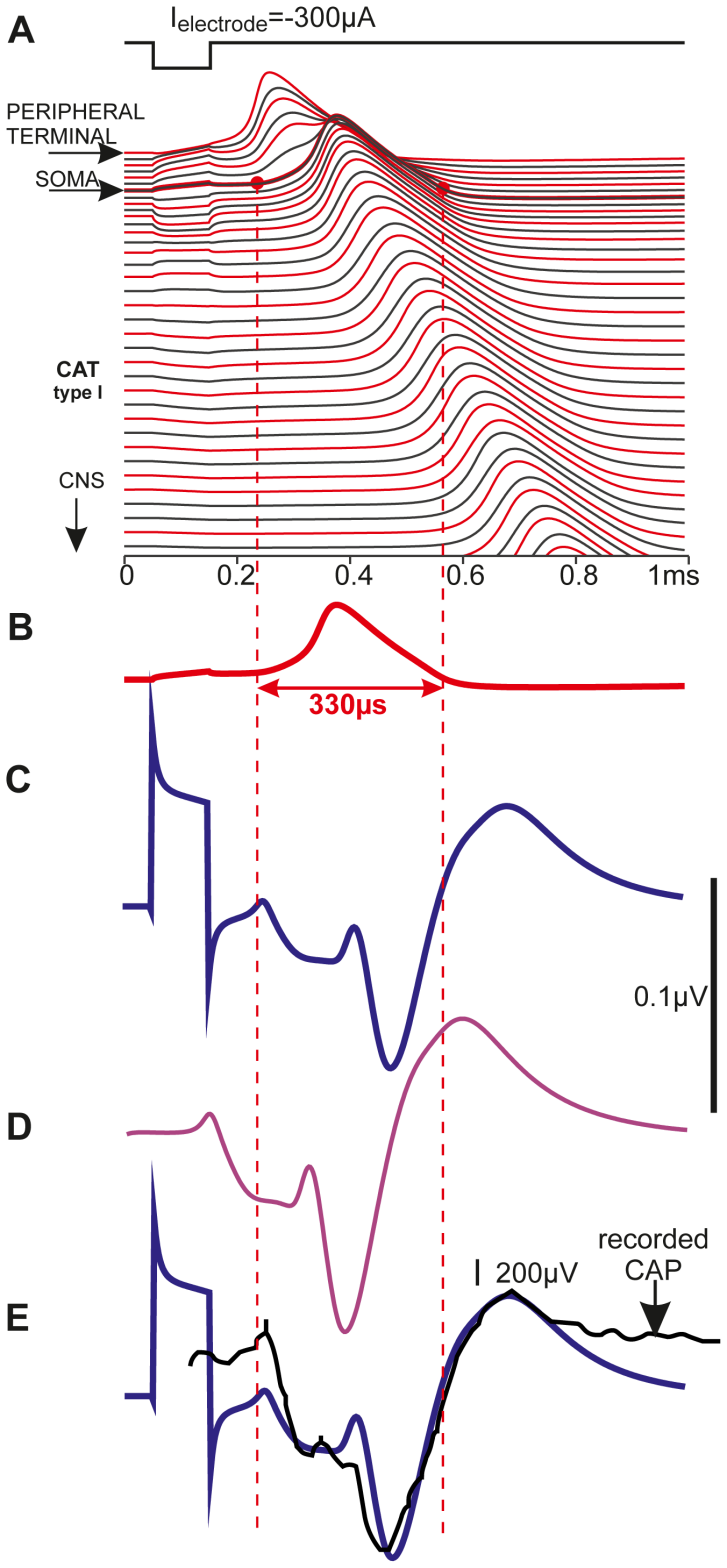
Temporal profiles of transmembrane voltages and extracellular potentials of an extracellularly stimulated feline type I cell. A small ball electrode simulates the situation of monopolar cathodic stimulation with a cochlear implant for a situation shown in Figure 1C. (A) During application of the 100 µs stimulus pulse the voltage across the membrane is influenced in each compartment. For this electrode placement the threshold is reached in the peripheral terminal and therefore the SGN excitation is similar to natural signaling. The transmembrane voltage lines, shifted vertically according to their distance along the neural path, show AP conductance; myelinated compartment responses in dark gray, compartments with voltage sensitive ion channels in red. (B) The short spike duration is demonstrated with the redrawn transmembrane voltage of the presomatic compartment. (C) Simulated extracellular potential for the position of the center of the stimulating electrode. (D) Simulated recorded signal for natural synaptic excitation, modeled as current injection into the first compartment (E) Simulated (blue, copy of C) and experimentally recorded (black) intracochlear voltage profiles generated with a cochlear implant show similar temporal characteristics although the simulated single cell activity is compared with a compound action potential recording. The black curve is redrawn from [51] (Figure 1, intracochlear recording, cathodic pulse −11.1 dB rel. 1 mA). Simulated situations correspond to scala tympani stimulation in the basal turn. Electrode position and neural path as in Figure 1C; homogeneous extracellular medium with extracellular resistivity of 300 Ohm.cm and other data as in [7].

In a critical model evaluation the computed AP duration was compared with experimental data. Applying the original Hodgkin Huxley dynamics at 27°C elsius for all active membranes (Figure 7 A,B) predicts quite short spike durations in our SGN model. But are the APs in the human and cat cochlear nerve really three times shorter than spikes of comparable thick axons in the central nervous system [24], [25], [49], [50]? As intracellular recordings are not available an answer to this question was found comparing simulated and recorded neural responses evoked by cochlear implant stimulation.

We simulated a cat SGN response to a cathodic 100 µs, 300 µA pulse from a spherical electrode (neural path and electrode position in the basal turn according to Figure 1C and Rattay et al. [7]. For this specific case, similar to natural excitation, a spike was initiated at the peripheral axon (Figure 7A). The spike is conducted with some delay before crossing the soma region. In the next step the transmembrane currents of the excited cell were modeled as local current sources and the sum of their contributions was computed for the position of the recording electrode which coincided with the stimulating electrode position [42]. The temporal characteristics of this voltage profile (Figure 7C) are similar to an intracochlear recording (Figure 7E) from Miller et al. [51] (weakest cathodic stimulus in their Fig. 1). The recorded signal is broader as it represents the temporarily shifted contributions from many excited SGNs whereas the computed signal results from a single cell response (compare scale bars in Figure7 C and E). Spike conduction in the peripheral and central axon caused separate minima in the simulated extracellular voltage profile (Figure 7C).

### Four phases in SGN signal transduction

**Figure 8:**
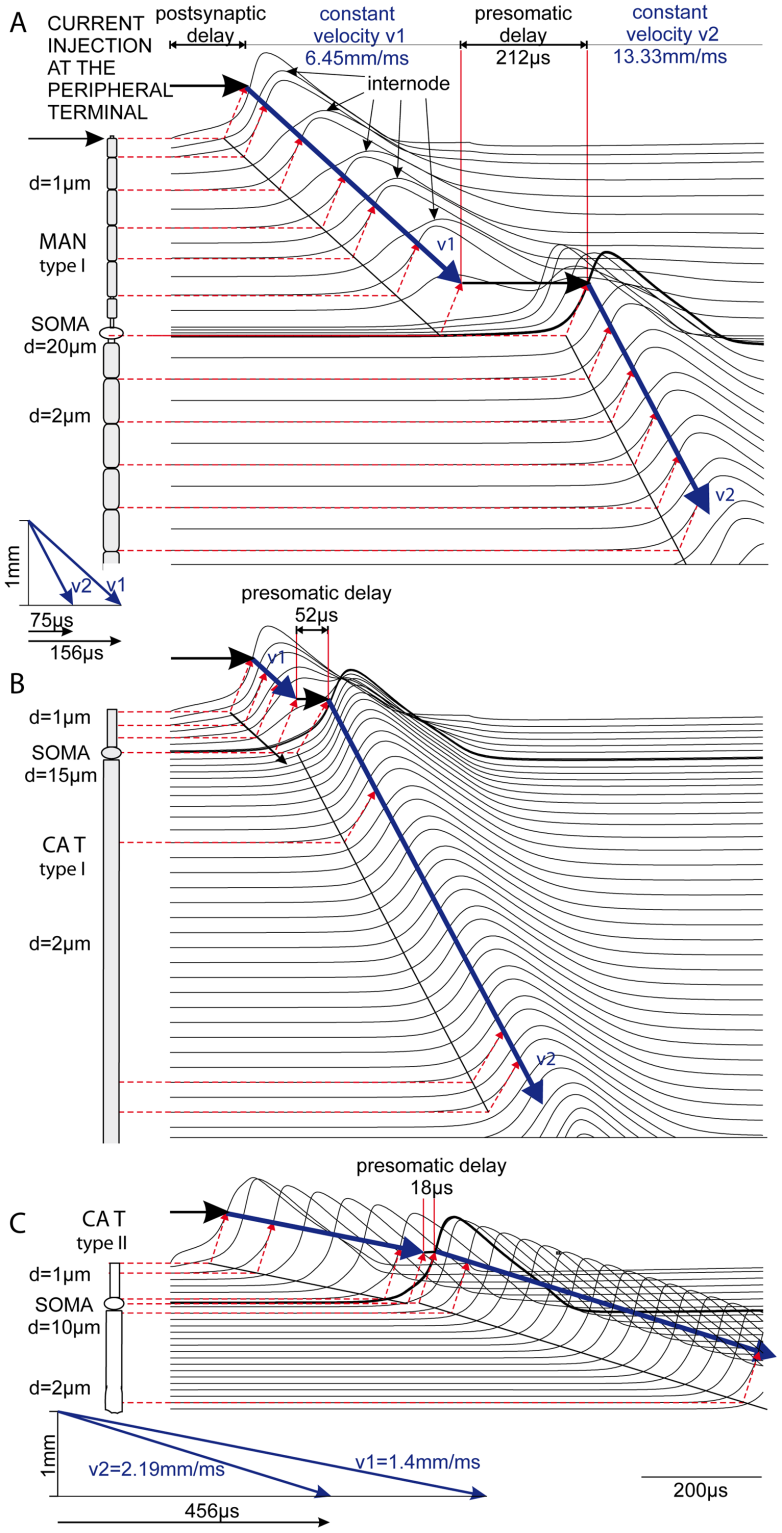
Simulated spike transduction in afferent cochlear neurons. Geometry of rectified SGNs (left, myelinated regions in gray) and transmembrane voltage profiles of the corresponding locations (right). For better comparison of phenomenological differences axon diameters and a peripheral terminal length of 10 µm are chosen to be the same in A–C. Spike initiation by a 0.5 ms pulse, 100 pA (A and B) and 500 pA for the non-myelinated case (C). Signal conduction with nearly constant velocities in the axons is indicated by the geometric construct with the broken red lines and thick blue velocity vectors. Note the attenuation of membrane voltage in the passive internodes and, most pronounced, in the presomatic region.

Spike transduction along SGNs can be divided into four characteristic phases. (i) Spike initiation at the peripheral terminal caused by either natural synaptic activation or exogenous current injection that results in a certain delay of excitation onset (postsynaptic delay t1). (ii) Axial current flow causes AP conduction in the peripheral process with velocity v1. There is only a small deviation from a constant v1 during the passage of the first node-internode segment. (iii) A considerable delay before the generation of the somatic spike is caused by the large soma capacitance that has to be loaded via axial current flow. Note the lower and long lasting voltage profiles in the presomatic compartments of Figure 8A indicated by vertical lines. (iv) Axial current flow from the soma into the central process again causes spike conduction with a velocity of v2.

The first characteristic phase, denoted as postsynaptic delay t1, is crucially influenced by the amplitude of an injected current pulse appropriate to simulate the synaptic SGN excitation by an IHC. For a 1 µm diameter myelinated peripheral process, a 100 pA square pulse caused a postsynaptic delay t1 = 133 µs for model data as illustrated in Figure 8A. Using a stimulus intensity comparable to recorded synaptic currents in rat, our simulations proposed a shorter postsynaptic delay in the order of 100 µs. The delay t1 does not include the neurotransmitter release from the hair cell and the diffusion across the synaptic cleft. In man and cat simulations demonstrated nearly the same short postsynaptic delays for type I SGNs (Figure 8A, B). In contrast to the assumptions in Figure 8C essentially longer postsynaptic delays are expected in type II cells. This phenomenon is analyzed below.

The second and fourth phases denote the spike conduction times t2 and t4 in either the peripheral or the central process. A linear distance-time relationship is shown by each of the blue velocity vectors of the propagating APs in Figure 8, demonstrating constant propagation velocities v1 and v2 in the peripheral and central axons, respectively. By evaluation of the shifted blue velocity vectors in the left graph in Figure 8A we found that the AP requires 156 µs to travel 1 mm in the 1 µm thick peripheral process and almost half of this time, 75 µs, in the central axon that has double the diameter. Using the computed data of the longer central process of the human type I cell (Fig. 8A, d2 = 2 µm, v2 = 13.33 mm/ms), this quite linear velocity-diameter relationship for the well wrapped myelinated fibers is fitted by
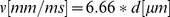
(1)wherein the factor 6.66 has the dimension 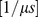 to match dimensional correctness.

The third phase, denoted by the presomatic delay t3, essentially depends on the axial current flow from the peripheral process required to load the somatic capacitance. Thus t3 is influenced by the size and degree of myelination at the somatic region. Reducing the soma diameter in 5 µm steps resulted in a decreased delay of 22 µs per step.

### Type I SGNs

The presomatic delay of 212 µs in human (Figure 8A) is shorter than previously reported by Rattay et al. [7]. The reason is a reduction of surface and capacity at the soma by a factor 4/9 when the diameter is decreased from 30 µm [7] to an average value of 20 µm which is close to the average size of our morphometric data [34]. The same effect occurs when the soma is further reduced to 15 µm which is the average value of cat type I cells. The higher number of myelin layers is even more influential since the somatic capacity decreases inversely with the number of covering membrane/myelin sheets. The human soma was simulated with 3 layers, and that of the cat with 13 layers. Thus the capacity of the myelinated cat soma is reduced by the factor (3/13)*(9/16)  = 0.13. Further arguments for the vastly short 52 µs presomatic delay in cat (Figure 8B) are given below.

Simulated spike propagation along a type I SGN is shown for man and cat in Figure 8A and B. In Figure 8B the vertical distances of the voltage profiles are markedly shorter since the internodes are assumed to be shorter in cat than in man, although the model fiber diameters are the same. In spite of shortening the internodal lengths, the calculated conduction velocities are almost the same in both cases. Reduction of the internodal length of the central process from 500 µm in man to 350 µm in cat causes a v2 increase <1% from 13.33 to 13.44 mm/ms. Such small influences of the internodal length on the AP conduction velocity in homogeneous myelinated axons are also expected in other modeling work [52].

The velocities v1 and v2 are diameter dependent, and they are evaluated with the same equation (1) for cat and man. However, the cat peripheral process has only 3 nodes of Ranvier and evaluation of the velocity/diameter ratio is less precise in comparison to human because uniformity of signal conduction is disturbed at both ends of this short axonal segment.

Spike conduction times for several diameters and different numbers of myelin layers enwrapping the somatic region (nmsoma) are listed in the upper part of Table 4. Note that for the postsynaptic delay a constant value of 100 µs is assumed for all cases. The presented values of the presomatic delay t3 are computed for a soma diameter of 20 µm in man. When the number of membrane layers is increased to 11 we calculated a 0.12 ms reduction of this delay compared to the unmyelinated situation with 1 layer. Since this delay t3 is also dependent on the diameter of the soma, the rising ratio of presomatic delay has been calculated for increasing the soma diameter with 1 µm steps. For standard type I neuron with 3 membrane layers around the soma (plasma membrane of SGN and 2 membranes of SGC) increasing the diameter to 21 µm resulted in a 4.3 µs increase of t3 (compare right column in Table 4). These linear relations hold for human soma diameters ranging from 10 to 30 µm when the soma is connected with a peripheral myelinated process with d1 = 1 µm in diameter. Doubled diameter d1 = 2 µm causes stronger axial currents and the presomatic delay decreases from 211+4.3 µs to 125+3.2 µs for the 20 µm standard soma with 3 layers (t3 column in the upper part of Table 4).

**Table 4:** Computed SGN spike conduction times with additional delay Δt per 1 µm soma diameter increase.

type I SGN										
d1	dsoma	d2 [µm]	nmsoma	t1	t2	t3	t4	t_total [ms]	Δt|dsoma+1 µm	
1	20	2	1	0.1	0.339	0.286	2.255	2.981	7.2 µs	man
1	20	2	3	0.1	0.339	0.211	2.255	2.906	4.3 µs	man
1	20	2	7	0.1	0.339	0.178	2.255	2.873	2.7 µs	man
1	20	2	11	0.1	0.339	0.166	2.255	2.861	2.2 µs	man
1.3	20	2.6	3	0.1	0.261	0.186	1.735	2.282	3.5 µs	man
1.3	20	2.6	11	0.1	0.261	0.148	1.735	2.244	1.5 µs	man
1.4	20	2.8	3	0.1	0.242	0.178	1.611	2.131	3.5 µs	man
1.5	20	3	3	0.1	0.226	0.169	1.504	1.998	3.4 µs	man
2	20	4	3	0.1	0.170	0.125	1.128	1.522	3.2 µs	man
1	15	2	13	0.1	0.120	0.052	1.125	1.397		cat

nonmyelinated type II SGN										
d1	dsoma	d2 [µm]	nmsoma	t1	t2	t3	t4	t_total [ms]		
0.73	10	1.3	1	0.8	0.624	0.043	8.771	10.238		cat
1	10	2	1	0.8	0.533	0.018	7.071	8.422		cat
0.65	10	1.4	1	0.8	1.869	0.043	16.903	19.615		man
1	10	2	1	0.8	1.507	0.018	14.142	16.467		man

myelinated type II SGN										
0.73	10	1.3	13	0.8	0.166	0.035	1.748	2.749		cat
0.65	10	1.4	11	0.8	0.497	0.083	3.061	4.441		man

d1 and d2 represent peripheral and central axon diameters; nmsoma denotes the number of surrounding single membrane layers in the soma region including the pre- and postsomatic segments. t1, t2, t3 and t4 denote postsynaptic delay, spike conduction time in the peripheral axon, presomatic delay and spike conduction time in the central axon, respectively. t_total  = t1+t2+t3+t4, Δt|dsoma+1 µm denotes the enlargement step of the presomatic delay when dsoma is 1 µm increased.

**Figure 9:**
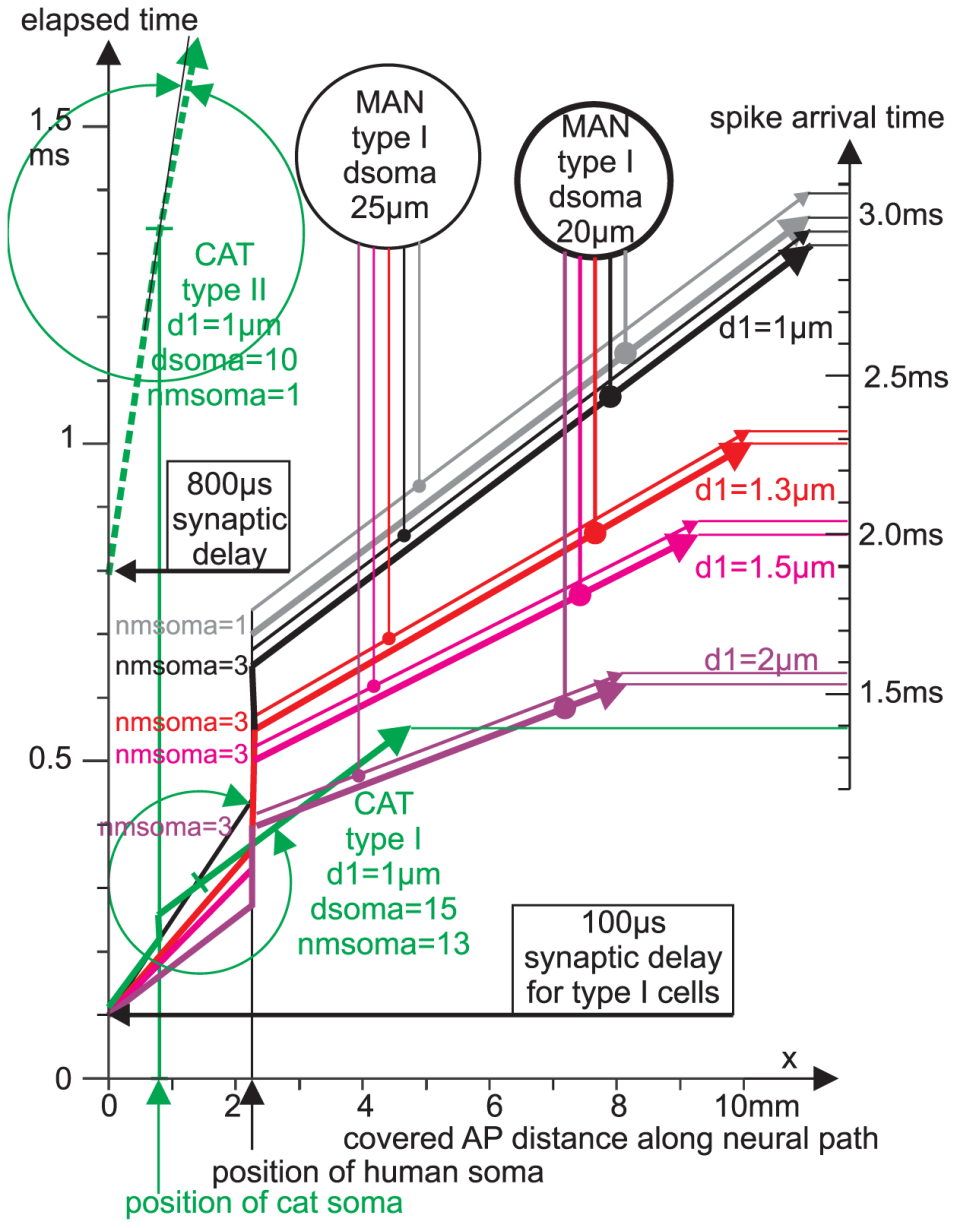
Main results of computed SGN conduction times. The diagram summarizes results from Table 4, indicating the impact of axon diameters, soma size and myelin on the arrival time under the assumption d2 = 2*d1 (central axon has doubled diameter of the peripheral process). The spike arrival time scale at the right side shows the total signal conduction time of type I cells for different axon diameters (marked by colors) for small somas (d = 20 µm, thick vectors) and large somas (d = 25 µm, thin vectors). All these cases are simulated with 3 sheets of membranes around the soma (nmsoma = 3) with the exception of the gray vectors (nmsoma = 1) which represent the slowest cases of type I cells. The fastest signal conduction in man (1.522 ms; d1 = 2 µm, dsoma = 20 µm; purple thick vector) is toped by the shorter cat type I cell (green vector). The main part of the figure shows the four phases in SGN signal transduction as distance – time diagrams. All vectors for type I cells start with the same synaptic delay of 100 µs. The lowest vector (purple, d1 = 2 µm) is shifted vertically (according to the presomatic delay) and flattened (because velocity v2 = 2*v1) when the spike arrives at the soma (black vertical arrow). All other vectors have the same characteristic shapes with individual slopes and individual shifts at soma. Note that the vertical time shift at the soma increases when axon diameter decreases. Increase of soma diameter causes an additional delay indicated by the vertical distance between corresponding thin and thick vectors. The slow AP conductance of unmyelinated type II cells (green dashed vector) is obvious: the short presomatic delay (beyond graphical resolution) cannot compensate the 800 µs lasting synaptic delay. The process diameter enlargement at the soma position (d2 = 2*d1) is not as effective as in the myelinated cases. Angles between v2 and v1 slopes (green circle arrows) point out small velocity changes (small velocity ratio v2/v1) in unmyelinated type II neurons (upper green circle) compared to type I ganglion cells (lower green circle arrows).

The main results of Table 4 are summarized in Figure 9, demonstrating how model parameters accelerate signal conduction of type I cells. Beside the neuron's path length, axon diameter increase has the largest impact on shortening the arrival time, followed by the increase of nmsoma and the decrease of soma diameter.

Therefore the greatest advantage of fast signaling in myelinated cat SGNs (in comparison to the human case) results from the shorter auditory nerve (15.81 mm in cat vs. 32.35 mm in man), followed by a smaller presomatic delay due to myelinization and smaller soma surfaces. Another anatomical difference in typical type I SGN favors the fast signaling in cat. The pre- as well as the postsomatic region in human are unmyelinated segments whereas in cat the soma is assumed to be between two nodes of Ranvier [7], (Figure 1A). Spike conductance in the rather long unmyelinated presomatic segment in human is slow and causes an additional delay which is shown in t3 (Table 4). This reduced velocity in unmyelinated axons explains why the 52 µs t3 delay for cat (last row in upper part of Table 4 for d1 = 1 µm, nmsoma = 13, dsoma = 15) is much shorter as expected by extrapolation of the 166 µs t3 value for man (Table 4, row 4 for d1 = 1 µm, nmsoma = 11, dsoma = 20) since a reduced soma diameter of 15 µm only causes a delay reduction of 5*2.2 = 11 µs.

### Type II SGNs

Spike conduction in unmyelinated fibers requires larger intracellular current flow because of missing the isolating internodal segments of myelinated axons. The lack of myelin sheets causes vastly larger capacities of the fiber resulting in essentially slower conduction velocities. Comparing myelinated and unmyelinated SGNs in cat, the greater somatic capacity in Figure 8B is more than compensated by the much stronger axial current flow supplied by presomatic regions. This current increase results from the uniform active membrane of the peripheral unmyelinated process which is more efficient for loading the soma capacity compared to the contribution from sparsely distributed active nodes of Ranvier in myelinated fibers. Consequently a negligibly small presomatic delay of 18 µs is predicted by our model evaluations (Figure 8C).

Still, in comparison with myelinated axons, conduction velocity is vastly slower. Spike conduction requires 456 µs and 719 µs for 1 mm in the peripheral and central process, respectively, resulting in v1 = 1.4 mm/ms and v2 = 2.19 mm/ms (blue vectors in Figure 8C, bottom). Theoretical investigations demand a quadratic velocity - diameter relationship for homogeneous fibers [20].

The relationship
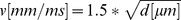
(2)with 1.5 of dimension
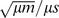
holds for the investigated non-myelinated fibers (Figure 8C) with a maximum deviation of 6%. This may be explained by irregularities in spike initiation at the beginning of each axonal section. Spike conduction times for the same process and soma diameters in cat and man are expected to differ only because of different process lengths (lower part of Table 4). Table 4 demonstrates that the slow afferent signaling from OHCs is based on a lack of myelin and, second, on an extreme long postsynaptic delay t1. Compare also myelinated vs. unmyelinated distance time diagrams in Figure 9.

As presented above, we have identified few myelinated type II cells in cat and man (Figure 2 A, D). In order to quantify the effect of myelination on type II cells the respective evaluated geometrical parameters were used to model a fully myelinated, OHC innervating neuron for cat and man. In cat, the myelinated type II neurons transmit an AP about 3.7 times faster compared to the unmyelinated standard case. In man, the corresponding neuronal structure transmits the electrical signal 4.5 times faster from the hair cell to the cochlear nucleus.

### Jitter and AP delay t1 are drastically reduced by high currents from ribbon synapses

**Figure 10:**
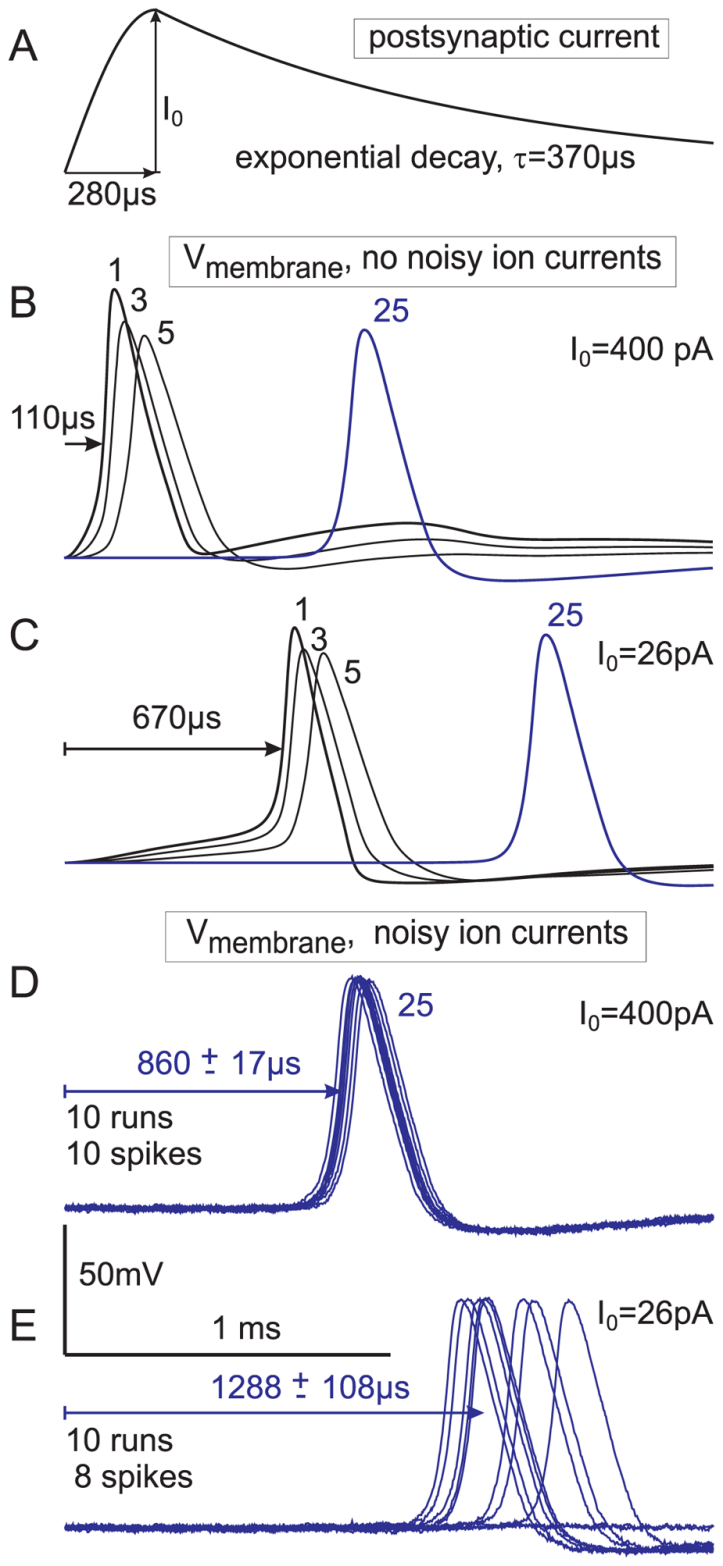
SGN response to strong and weak synaptic stimuli. (A) Postsynaptic currents from rat experiments are characterized by amplitude, time to peak and time constant for decay. (B–E) responses of a type I SGN with parameters from Figure 8A are shown for compartments # 1, 3, 5 and 25. Reduction from a typical synaptic current amplitude (B) to threshold (C) caused an essentially longer delay. Including ion current fluctuations (noisy membrane current model, [7]) in all compartments with active channels resulted in sharply synchronized responses for strong stimulation (D) and in late responses with large jitter (E). Compartment 25 is the fifth postsomatic node of Ranvier in the central process and represents the main part of the expected jitter at the proximal axon ending.

The first 10 µm long compartment of our model neuron simulates the short dendritic terminal that connects a type I SGN with an IHC [6], [7], [10]. In Figure 8 A,B current injection to this segment was a 100 pA, 0.5 ms pulse, which is a rather weak stimulus in comparison to currents from IHC ribbon synapses with an average amplitude I_0_ of ^∼^400 pA, 280 µs for time to peak and a decay time constant τ of 370 µs as recorded in adult P20 and P60 rats (Figure 3 in [29]). Applying stimuli of the recorded shape (Figure 10A) and intensity immediately initiated spikes for the standard human type I SGN geometry (Figure 10B). The fact that the spike in the first compartment almost returned to resting potential when the synaptic current reached its peak time (Figure 10A,B) is a consequence of the notably strong stimulus current which exceeds the threshold (Figure 10C) by a factor of at least 15. Note the increased spike amplitude of compartment 1 and even of compartment 3 in Figure 10B.

Synchrony in spiking is disturbed by ion current fluctuations in each active compartment with an intensity that was assumed to be proportional to the square root of the number of sodium channels involved [7]. This noisy ion current approach demonstrated a small jitter for strong stimuli (Figure 10D) but a large disturbance of synchrony for stimuli in the lower suprathreshold regime (Figure 10E). Beside loss of spikes in the order of 20% (Figure 10E) an increase of signal transduction time of about 500 µs can be expected when type I neurons would be stimulated with I_0_ = 26 pA.

In contrast to type I cells, the weak stimulus current I_0_ = 26 pA seems to be typical for afferents from OHCs [30], [53]. Whereas type I cells contact single IHCs, type II terminals arborize extensively among OHCs. Seven or even many more synaptic OHC contacts in a region of several hundred micrometers are assumed to be the average for a type II cell [53], [54]. Because of a lack of data we have not simulated one of the various geometries of the distal terminals of type II cells. The large length constant in the order of 1 mm as reported Weisz et al.[30] supported our assumption to simulate this structure again as a single compartment and to use for a rough approach the same excitation characteristics as for the type I cell in Figure 10.

Despite their greater pool of synaptic inputs, the frequency of synaptic events in type II afferents is assumed to be about one tenth of that observed in type I cells [53]. The spatial distribution of synaptic contacts within the terminal causes an additional irregular delay component in t1 for the signal propagation within this segment. Three new components of t1 for type II cells should be involved in the model approach: (i) an additional delay of 428 µs corresponding to the time difference for the spike arrival times in compartment 25 according to cases D and E of Figure 10, (ii) a large jitter and (iii) a further delaying component representing the increased loss of effective synaptic current as consequence of larger axial currents in unmyelinated axons (larger stimulus in Figure 8C than in Figure 8B). The total t1 delay can be estimated with 600-1000 µs. A t1 value of 800 µs is used in Table 4 for all type II SGNs.

## Discussion

A vast majority of human SGNs (96.35%) are embedded in a continuous, honeycomb structure formed by satellite glial cells [16] representing the standard case of type I cells. Thus, each unmyelinated type I cell body is encapsulated by at least one cellular layer of a satellite glial cell [14] resulting in two additional membranes. This micro-anatomical peculiarity is interpreted as nmsoma = 3 for our standard cochlear type I neuron. The assumed nmsoma = 11 for the myelinated case is based on 4 and 17 (mean = 10.5) myelin layers enwrapping the cell bodies [14]. This group and Arnold [55] report that the myelin sheaths were observed to be compact, loose or semicompact, although the loose form was most common.

The process diameter relationship d2 = 2 * d1 precisely holds for each analyzed human specimen (n = 3) everywhere along the cochlea spiral. This relationship may also be valid for type II neurons even though the respective diameters (of only one cell) were determined to be half the size compared to type I neurons. Interestingly, the highest density of type II neurons was found within the middle turn of the analyzed specimens where the majority of phonational frequencies are situated [48], [56].

Based on morphometric data, degree of myelination and postsynaptic hair cell currents, we used a biophysical model to simulate and compare excitation profiles and spike propagation in human and feline SGNs.

### Spike duration

In contrast to long lasting spikes of a SGN mouse model [21] the AP duration of 1/3 ms of our model was shown to be in agreement with temporal properties of intracochlear recordings (Figure 7E). Such short APs are known from peripheral nerve models for myelinated axons in frog, rat and rabbit [20], [57]–[61]. AP durations longer than 1 ms are typical for excitable membranes in the central nervous systems [24], [25], [49], [50]. Shorter spikes are reported from octopus cells in the ventral cochlear nucleus [26], but the SGN spikes are even shorter. The time to peak and the duration of an AP depend on the types of ion channels, their kinetics and densities, the membrane capacitance and other parameters. Although many relevant details are known for mammalian SGN membranes, as yet there is no accurate cable model available which matches the AP shape of type I cells. In our model we fitted temporal spike characteristics by changing two parameters of the original HH model, temperature and ion channel density. Another approach for a HH type model is to assume that channels expressed on soma are identical, but differ in density in the processes. The channel densities can be fitted to *in vivo* recordings [62]. An appropriate short and rapidly increasing AP as simulated with our model is a key element for the quick signal conduction in the afferent part of the auditory nerve. The short spikes and the fit of the formula for axon conduction velocity of type I cells (Eq. 1) to experimental findings on peripheral myelinated axons (see below) underlines closer electrophysiological affinity of SGNs to the peripheral nerve system than to the central one.

### Simulated conduction times correlate with ABR

The simulated spike conduction times for afferent signal transmission can be evaluated with auditory brainstem response (ABR) data as time difference between the peaks of wave I and wave III. Esteves et al. [63] reported corresponding ABR interpeak times of 2.13±0.14 ms (n = 120 ears) in normal hearing human subjects. Van den Honert and Stypulkowski [64] analyzed ABR recordings of 10 normal hearing cats and reported a mean signal latency between waves I and III of 1.41±0.1 ms. According to our model, the corresponding values in Table 4 are 2.13 ms for a peripheral process diameter d1 = 1.4 µm (man) and 1.40 ms for d1 = 1 µm (cat). Whereas the cat data match almost perfectly, our systematic morphometric evaluation from human specimens rely on the d1 = 1.3 µm case which predicts a 150 µs longer conduction time of 2.28 ms.

Closer examination of the cat data (Table 3) shows that the systematic diameter evaluation in cat deviates from the d2/d1 = 2 relation used in Table 4. Reducing d2 from 2 µm to 1.8 µm demands for a 10% reduction of velocity v2 (Eq. 2) and a 10% increase of t4 according to Table 4, leading to an additional delay of 112 µs. A possible explanation for this discrepancy between spike conduction times and ABR interpeak times may be found with the help of Figure 7 where the conducted spike is recorded within the cochlea. Although the recording electrode is close to the thin peripheral process (Figure 1C) the main contribution in the recorded signal appears when the spike passes the soma region (Figure 7). This activity should contribute more to the peak I in ABR recordings than the excitation of the peripheral axon. ABR peak III should be generated when the spike arrives at the increased surface of excited membranes in the branching central SGN terminals causing synaptic activation in the cochlear nuclei.

Electrically evoked ABRs from cochlear implantees were analyzed recently [65]. Poststimulus latencies of peak III were shorter for anodic pulses and they clustered at about 2 ms. In most of the reported cases spike initiation can be assumed within the central axon at a site close to the soma. This 2 ms delay which is similar to the interpeak time III - I recorded in healthy people [63] supports a hypothesis derived from Figure 7D where it is seen that the second peak exceeds the contribution of the thin peripheral process. This effect is assumed to be more pronounced in human type I cells because of the higher current flux across the active membrane in the larger presomatic compartment. Therefore, peak I in healthy people may represent the large sodium current influx when the spike enters the nonmyelinated presomatic region.

### How myelin accelerates signaling

The examples in Figure 8 and Table 4 suggest two types of acceleration of signal conduction by myelin, namely at the axons and in the soma region. The values t2 and t4 in the upper and lower part of Table 4 demonstrate the rapid conduction of myelinated fibers compared to the non-myelinated. The factor 6.66 for the linear relation between axon diameter and simulated velocity (Eq. 1) is quite close to 6.57, which is the corresponding scaling factor for small diameter myelinated axons as reported by Boyd and Kalu [66], who found an average value of 4.6 for v/D. Incorporating an inner/outer fiber diameter ratio d/D = 0.7, the equivalent scaling factor is 4.6/0.7 = 6.57.

Reduction of myelin layers and the markedly bigger diameter of the soma are the reason for a notable capacity enlargement resulting in a distinct presomatic delay for bipolar cochlear neurons in man. This delay is smallest for non-myelinated cells with a small diameter ratio of soma / peripheral process, e.g. 18 µs for type II cells (t3 in the lower part of Table 4 and Figure 8C) but increases for human type I cells to 286 µs (d1 = 1 µm, dsoma = 20 µm, nmsoma = 1; Table 4) and even higher delays are expected for large somata (Figure 9)[7]. According to observations of Spoendlin [10] there is a single node of Ranvier in the vicinity of the cat soma. Biophysical modeling demonstrates the need for a considerably longer active membrane length of the presomatic segment in cases of poorly myelinated somata (Figure 4B) in order to provide enough inneraxonal current to load the capacity of the cell membrane. The length of this presomatic nonmyelinated segment was not systematically determined, but it is always considerably longer than a node of Ranvier, e.g. Ota and Kimura [14] measured 5–26 µm long presomatic segments in human type I cells.

Our auditory nerve model confirmed that the conduction velocity of a uniformly myelinated axon is proportional to the diameter but it is rather independent from the internodal length [52]. However, signal amplitude is smaller in the internode than in the voltage amplifying node. This phenomenon is especially evident in human type I cells at the last internode before the presomatic section. In contrast to the myelinated cat soma, the weak inneraxonal current causes a remarkable presomatic delay and endangers signal propagation. Two or more cell bodies of human SGNs sometimes comprise a common insulation by glial cells. Within such a cluster a close contact of somatic cell membranes is evident. Tylstedt and Rask-Andersen [67] speculate whether unique formations between such human SGCs may constitute interactive electrotonic or ephaptic transmission pathways. These may increase neural synchrony and signal acuity related to the coding of speech. This hypothesis will be tested in a forthcoming paper as well as our suggestion that clusters could also work as filters in order to suppress spontaneous spikes without acoustic signal information.

### Synaptic hair cell currents

Another surprising fact predicted by the biophysical model can be deduced from the large stimulus of ^∼^400 pA recently recorded from the IHC ribbon synapse [29] which is in accordance with our simulations of about 15 times the threshold current of typical type I SGNs. Such strong stimuli cause an accelerated spike propagation in the peripheral terminal via increased axial current leading to an extremely short postsynaptic delay t1 of ^∼^100 µs. Weak synaptic stimulation, e.g. 26 pA, as found in type II cells [30], [53] causes a longer delay t1 in the order of 1 ms but also a large AP jitter that disturbs synchrony of the firing SGNs. This excess of stimulus energy is obviously important for quick and precise signaling in type I cells.

### Significance for natural hearing and cochlear implants

The quite constant diameter ratio of 1/2 for peripheral/central processes and the similarity in distinctive myelination of human type I SGN guarantees constant spike conduction times in each frequency region of the cochlea. Taking into account the coiled symmetry of the cochlea, uniform signal conduction times are most likely for APs triggered within the basal turn as well as for signals originating from the middle portion. In the low frequency region, increasing conduction times can be expected [1], [2] due to varying lengths of the peripheral processes. However, this continuously rising time delay for signals from the apical region can be assumed to have insignificant effects due to rather constant length of SGNs responsible for frequencies of one octave. This neural architecture maintains most of the temporal information of acoustic signals and allows phase locking up to 4 kHz in the spiking patterns of higher auditory centers of the brain ([1], [2] Figure 4B). Contrary to natural hearing, cochlea implants initiate spikes at different sites along SGNs [5]–[7] which depends on the degree of preservation of cochlear neurons [68]–[70]. The resulting temporal mismatch within a population of excited cells is mainly caused by simultaneous AP initiations in peripheral and central axons. As an example, the presented biophysical model predicts the lower threshold in cat for cathodic pulses but in man for anodic ones [7], which was assumed to be the same in both species but model results have been confirmed by implantees [71]. This study has shown that the differences between the cat and human SGNs, for example a considerably longer human presomatic delay, should be incorporated into auditory nerve models that rely on the data from the cat when investigating speech processing strategies for cochlea implant recipients.
